# High-throughput processing and normalization of one-color microarrays for transcriptional meta-analyses

**DOI:** 10.1186/1471-2105-12-S10-S2

**Published:** 2011-10-18

**Authors:** Mikhail G Dozmorov, Jonathan D Wren

**Affiliations:** 1 Arthritis and Clinical Immunology Research Program, Oklahoma Medical Research Foundation; 825 N.E. 13th Street, Oklahoma City, Oklahoma 73104-5005, USA

## Abstract

**Background:**

Microarray experiments are becoming increasingly common in biomedical research, as is their deposition in publicly accessible repositories, such as Gene Expression Omnibus (GEO). As such, there has been a surge in interest to use this microarray data for meta-analytic approaches, whether to increase sample size for a more powerful analysis of a specific disease (e.g. lung cancer) or to re-examine experiments for reasons different than those examined in the initial, publishing study that generated them. For the average biomedical researcher, there are a number of practical barriers to conducting such meta-analyses such as manually aggregating, filtering and formatting the data. Methods to automatically process large repositories of microarray data into a standardized, directly comparable format will enable easier and more reliable access to microarray data to conduct meta-analyses.

**Methods:**

We present a straightforward, simple but robust against potential outliers method for automatic quality control and pre-processing of tens of thousands of single-channel microarray data files. GEO GDS files are quality checked by comparing parametric distributions and quantile normalized to enable direct comparison of expression level for subsequent meta-analyses.

**Results:**

13,000 human 1-color experiments were processed to create a single gene expression matrix that subsets can be extracted from to conduct meta-analyses. Interestingly, we found that when conducting a global meta-analysis of gene-gene co-expression patterns across all 13,000 experiments to predict gene function, normalization had minimal improvement over using the raw data.

**Conclusions:**

Normalization of microarray data appears to be of minimal importance on analyses based on co-expression patterns when the sample size is on the order of thousands microarray datasets. Smaller subsets, however, are more prone to aberrations and artefacts, and effective means of automating normalization procedures not only empowers meta-analytic approaches, but aids in reproducibility by providing a standard way of approaching the problem.

Data availability: matrix containing normalized expression of 20,813 genes across 13,000 experiments is available for download at . Source code for GDS files pre-processing is available from the authors upon request.

## Background

Using microarrays for hypothesis generation and validation has become routine in virtually every area of biomedical research. However, the majority of generated data is underutilized since publications reporting microarray results often focus on a small subset of the results they feel are most relevant to their research focus, even if other interesting observations may be present in the data. Thanks to the Minimum Information About a Microarray Experiment (MIAME) [1] to standardize descriptions and reproducibility, along with the requirement imposed by most journals to make microarray data publicly accessible, this wealth of data is accessible to the community. Microarray data repositories, such as Gene Expression Omnibus (GEO) [2], ArrayExpress [3], Stanford Microarray Database [4] contain growing amounts of gene expression data from multiple biological organisms and treatments. For example, besides just storing microarray data, GEO also provides a simple web interface to analyze and compare individual GDS (GEO Data Set) files. ArrayExpress also provides a means to access its database via ExpressionProfiler software [5] and with Bioconductor R package for subsequent analysis. However, the ability to conduct a large-scale meta-analysis focused on a specific condition or disease, is not provided and neither is it trivial to compile a list of datasets associated with such conditions that are directly comparable. One of the chief concerns is the heterogeneity among probe design within microarray platforms, laboratory variations, and methods of data pre-processing.

Whether the meta-analysis is focused on specific experimental types [6,7] or is aimed at a global assessment of gene expression patterns across all experiments [8-10], the major hurdle is that it heavily depends on the quality of the underlying data. For example, if a probe on one platform hybridizes to a much longer transcript than on another platform, the probe intensity will appear constitutively higher and direct comparisons will suggest differential expression. The accuracy and reproducibility of commonly used microarray platforms has been hotly debated with results ranging from relatively discouraging [11,12] to promising [13,14]. A multi-center consortium, MicroArray Quality Control (MAQC) performed independent assessment of gene expression data reproducibility and found results to be very optimistic [15]. Furthermore, MAQC II analysis confirmed biological differences as the most readily detected value [14,16]. However, a human factor (i.e. people conducting identification of biological differences) is one of the most important pieces on the analysis [16]. As such, detection of true biological differences require a comprehensive method of diverse microarray data integration conducted with an understanding of the underlying technical and biological issues.

Gene nomenclature poses another serious problem in comparison of different microarray platforms. Gene identifiers, such as GenBank, Illumina IDs, Affymetrix IDs have different underlying annotations and are not directly comparable. Several attempts have been made to overcome those hurdles. Two tools, List of lists-annotated (LOLA) [17] and List to List (L2L) [18] were created to compare gene lists against microarray data from different platforms, different nomenclatures, or even different organisms. However, these tools rely on published data and need to be manually curated. A cancer-oriented database, ONCOMINE [19], was developed for queuing gene expression profiles in different tumor types and tissues. CellMontage allows users to correlate a custom pre-processed gene expression dataset with GEO datasets grouped by platforms [20]. M2DB is a curated microarray database which is designed for easy quality control and retrieval of raw and normalized microarray data [21]. A major drawback of these tools is that they require different input format and produce different results often biased towards particular tissue, platform (CellMontage, M2DB) and/or disease (Oncomine).

Several papers have been published that thoroughly outline the challenges, opportunities and recommendations for standardization of microarray meta-analysis, discussing the benefits and pitfalls, and comparing methods for data processing [10,22-26]. Rather than develop a different method, the work reported here is based on a combination of the recommendations reported in these studies (e.g., mapping probe IDs to a unified Entrez identifier). In addition, we consider fundamental properties of microarray data distributions [27,28] to standardize different experimental data for meta-analysis. We present a straightforward method of extracting gene expression data from publicly available datasets, performing quality control of the data, comprehensively mapping it to Entrez Gene IDs and creating a gene expression matrix from multiple experiments. Our method is based on the use of intrinsic properties of microarray data, adjusted by quantile normalization to unify distribution of gene expression across diverse experiments and to accurately determine a level of technical noise. We compile a gene expression matrix from 13,000 human 1-color microarray experiments, establish noise level and make expression values comparable across datasets. We showed our pre-processing steps increase recall and precision for prediction analysis.

## Methods

### Obtaining one-color microarray data

GEO Datasets (GDS) files were downloaded from ftp://ftp.ncbi.nih.gov/pub/geo/DATA/SOFT/GDS/ and uncompressed from .gz compression format. Files were selected for processing if the following fields were dataset_sample_organism=“homo sapiens”, dataset_type=”nucleotide” or “gene expression”, dataset_channel_count=”single” or “1”, and dataset_value_type=”count”. This ensures only raw data from one-channel human microarray samples were processed.

### Probe mapping

Probe mapping was done using a relational database, assigning unique Entrez ID identifiers based on gene names and accession numbers downloaded from NCBI (ftp://ftp.ncbi.nih.gov/gene/DATA/GENE_INFO/Mammalia/Homo_sapiens.gene_info.gz). Unmapped probes were stored in a file and examined if any of the platforms was unmapped due to absence of its mapping data in the database. Each technological platform has unique set of probes targeting different gene regions. Moreover, some probes recognize particular isoforms of the same genes, such as implemented in Affymetrix and Illumina platforms. Affymetrix uses extensions to its unique IDs, such as “_at” indicating a probe recognizing a unique gene isoform. “_s_at” extension indicates a probe can recognize multiple isoforms of the same gene. Illumina flags its IDs by “I” (a probe recognizes a single isoform) and “A” (a probe recognizing all isoforms). Due to aforementioned problems of linking diverse manufacturer’s IDs to unique gene identifiers it is logical to use probes recognizing all isoforms of a given gene. Such probes should have maximum expression value relative to other probes that only recognize individual isoforms of the same gene. Therefore, the maximum expression value was selected from multiple probes targeting the same gene.

### Data pre-processing

Basic parameters were calculated for each dataset, namely, mean and median. Datasets with mean or median equal to 0 were excluded, as well as the datasets with mean to median ratio equal to or less than 1 (see Results for explanation). Data for each experiment were sorted and distribution of expression values around maximum was examined. Due to technological imperfections some genes demonstrate extreme saturated measurements at the high end of expression spectrum. Such expression values would negatively influence the following quantile normalization step by introducing artefacts, and should be adjusted. Overall, no more than 0.1% of maximum expression values showed extreme measurements. For each experiment, 0.1% of genes with highest expression values were selected, and a minimum expression value (flooring value) among them was identified. Expression of these genes was replaced with this flooring value. Each experimental dataset was then adjusted to fit within 0 - 10,000 range.

Low expression values in each microarray dataset constitute technical noise that can be approximated with normal distribution [27]. To make distributions of different datasets equal the data fit within 0-10,000 range were quantile-normalized [29]. Briefly, quantile normalization replaces distributions of each dataset with an average distribution, calculated from an average of sorted expression values across all datasets. This step does not alter the data structure (see Results for explanation) but makes it possible to define common noise threshold and directly compare expression level across the whole matrix.

### Validating data preprocessing steps

We explored the data structure in 1) The matrix containing raw expression values; 2) matrix containing scaled to 0-10,000 range data; and 3) matrix containing scaled and quantile normalized data. We performed gene ontology prediction analysis [8] and calculated precision/recall based on the number of correctly predicted gene ontologies (Equations 1 and 2). We show our results in a form of F-measure, a test for accuracy that considers both precision and recall (Equation 3).

*Equation 1*: 	Recall = TP/(TP+FN)

*Equation 2*: 	Precision = TP/(TP+FP)

*Equation 3*: 	F-measure = 2*Precision*Recall/(Precision+Recall).

## Results

A total of 2,325 GDS files were downloaded, out of which 587 contained raw gene expression data from human single-channel microarrays. Probe identifiers were mapped to Entrez IDs, totaling 20,814 genes.

### Quality control: mean/median ratio

**Figure 1:**
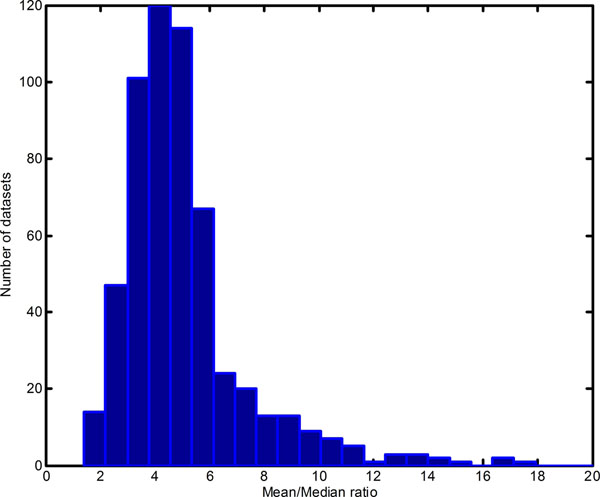
**Frequency histogram of mean/median ratio distribution of datasets used for processing.** Datasets with MM ratio less than 1.2 were excluded.

Single-channel microarray data follows well-defined distribution that can be approximated with a lognormal model [27,28]. Genes expressed below noise level form a pronounced normal distribution at low expression levels while genes expressed above noise are spread across the whole spectrum of expression. These properties of microarray data distribution dictate that its mean and median parameters can’t be equal, whereby a median should be always smaller than a mean. Thus, the mean/median ratio in a dataset should always be more than 1. Investigation of Mean/Median ratio (MM ratio) in all datasets proved this to be the case for majority of datasets. Median value for MM ratio was 4.55 (Figure 1). Seven datasets (139 experiments total) with MM ratio less than or equal to 1.2 were removed as suspects for bad quality data. A total of 577 datasets with 13,000 experiments were used for further analysis.

### Data re-scaling

While remaining datasets did not contain negative values, raw expression data distribution may show inconsistent intensities at very high expression level [30]. Manual inspection of all datasets for possible outliers identified on average 0.1% (~20 genes) of the top expression values showing extreme measurements. Such extreme measurements would distort data rescaling in unpredictable ways. To minimize the impact of such outliers they were set to their minimum value (floored). This step included selection of top 0.1% of genes with highest expression level in each unprocessed experiment, identifying their minimum and setting them to that minimum. This change on 0.1% expression values did not affect data distribution (data not shown). The resulting matrix of 13,000 experiments containing expression values for 20,814 genes was scaled to 0-10,000 range.

### Quantile normalization

**Figure 2:**
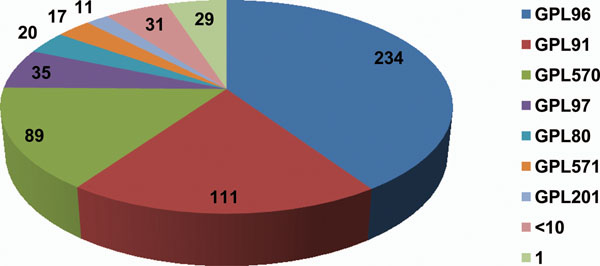
**Platforms and number of datasets used in the current study.** A total of 43 platforms were used, which comprised 577 datasets and 13,000 experiments.

Gene expression matrix from 13,000 experiments contained data from different experimental platforms. Affymetrix platforms delivered highest number of datasets, with 234 datasets done on Affymetrix Human Genome U133A Array (GPL96) followed by 111 datasets on Affymetrix Human Genome U95A Array (GPL91) platform (Figure 2).

**Figure 3:**
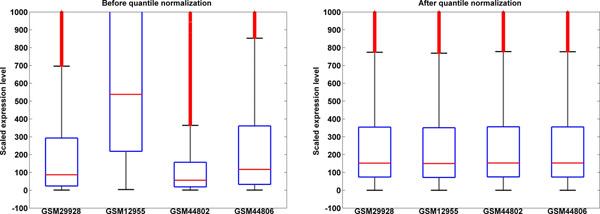
**Box-and-whisker plots of data distribution in a sample dataset before (A) and after (B) quantile normalization.** X axis – dataset names, Y axis – expression range, only values in 0-1,000 range shown for clarity.

Even though different platforms yield comparable experimental results [15], the data from different platforms produced different distributions. That is, the parameters of the lognormal model that can be fitted to them [27,28] differ (Figure 3A). To make them comparable we applied quantile normalization [29]. Quantile normalization only rescales data distributions to make them fit to an average distribution calculated from all datasets. An example of data before and after normalization is shown in Figure 3.

### Distribution after quantile normalization

**Figure 4:**
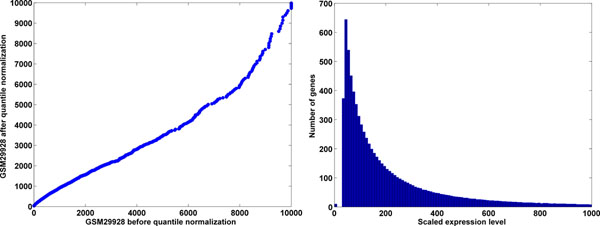
**Data distribution before and after quantile normalization.** Example of expression changes in a dataset before and after quantile normalization, and a frequency histogram of an average distribution fit to all datasets. A) Data from a sample dataset plotted before (X-axis) vs. after (Y-axis) quantile normalization. No major distortions were observed, quantile normalization introduced only transitional rescaling to the data; B) An average distribution fitted to all datasets. This distribution allows setting global noise threshold and directly comparing expression levels across the datasets.

Data before and after quantile normalization correlated with each other (Figure 4A), R^2^=0.99. Quantile-normalized data from all experiments has the same distribution (Figure 4B) and preserves the rank-order of genes by expression level in each dataset. A normal distribution was fitted around the peak of low expressed genes and its parameters were determined. Mean was determined to be 36 and standard deviation (SD) was 26. As such, the noise level threshold, commonly defined as 3 SD above mean was determined to be 114, above which the level of gene expression can be detected with 99% accuracy. Thus, genes with expression values >114 in the resulting matrix can be considered expressed with p < 0.01 in any given experiment.

### Testing the effect of normalization on predicting gene function

It is hard to empirically demonstrate the superiority of one normalization approach over another. It is, however, important to know if your methods of normalization had a positive effect. Recently, we described a means of using a global microarray meta-analysis of gene-gene co-expression patterns to predict gene function [8]. The basis for this is successful grouping of gene-gene co-expression patterns from heterogeneous datasets to identify gene pairs whose co-expression is strongly correlated regardless of the experimental condition. Genes that have strong correlations across datasets tend to share similar biological functions. We found the top 20 co-expressed genes (e.g., as judged by the strongest Pearson’s correlations) tend to accurately predict the GO categories of any given gene of interest [8]. As we report in a related study [31], we find that the predictiveness of these genes rapidly declines the more that are taken for analysis, suggesting the best accuracy will result from effectively identifying and grouping the most correlated genes. Technical noise, however, should affect the cohesiveness of pairings and, consequently, the precision by which we can predict biological function. We hypothesized that raw data should produce more erroneous predictions because it is noisier and contains distortions within the data distributions that should affect accurate measurement. Thus, if the normalization scheme is effective and biologically relevant, it should increase the accuracy by which gene function can be predicted on the basis of global co-expression patterns in raw data.

To test this, we conducted a global meta-analysis to predict GO categories. 16,140 genes with known GO categories were used for analysis. The most highly correlated genes in terms of their co-expression patterns across all 13,000 microarrays were identified using metrics described in a related work [31]. For each gene, its top 20 genes were used to predict it’s GO category. The results were compared against its annotated GO category, looking for the fraction of predicted categories that were exact matches to the annotated categories. In this analysis we wanted to identify the effects on both precision (i.e., ability to correctly predict GO categories) and recall (i.e., ability to find as many known annotations as possible). As such, we used F-measure (defined as (precision+recall)/2)) to estimate the effect of pre-processing steps and normalization on predicting gene functions.

We compared predictions using the raw data, data that was scaled and outliers eliminated as described, and data fully normalized as described herein. We found using the raw data, the F-measure was 0.114. Scaling the data to a common 0-10,000 range increased the F-measure to 0.125, and using quantile normalized data allowed us to reach an F-measure of 0.130. These results demonstrate that each pre-processing step increases the ability of the global meta-analysis to identify biologically relevant patterns of co-expression.

## Discussion

One enabling factor for high-throughput data standardization is that the input data files should have a well-defined structure for mapping data elements. Specifically, within GEO, the GDS (GEO dataset) files work best for that purpose, since they are reassembled by GEO staff and stored in text files with information fields. GDS files are standardized versions of the originally submitted GSE (GEO series) files. GSE datasets lack a standardized structure and will require manual reassembly, which currently lags the production of such datasets and is a reason others have chosen to exclude them as well [10], [32].

One of the primary challenges in standardizing datasets is choosing an appropriate identifier for each gene. Fortunately, many tools exist to convert microarray IDs between database probe names and among the more developed are DAVID [33] and RESOURCERER [30]. One dedicated effort, AILUN, attempted to map all GEO IDs to a unique identifier [34]. Our solution here was to try to map to a common, widely used identifier, the Entrez gene IDs [35] (superseding LocusLink) as defined by NCBI. Entrez’s “one gene – one ID” concept suits well for the purpose of bringing expression values from multiple probes targeting one gene to a single placeholder, the only drawback being that some probes on lesser used platforms may not map to a gene ID. The number of such experimental probes varies significantly from platform to platform. Ultimately, next-generation sequencing will provide an even bigger challenge for meta-analysis, as many sequences identified will not have existing identifiers.

Single-channel microarray data have been shown to exhibit a well-defined distribution of their values [27,28]. A frequency histogram of low-expressed values shows a peak that approximates a normal distribution, the parameters of which serve as a platform-defined (and platform-dependent) noise threshold. Expressed probes are those on the rightmost tail of the distribution -the highly expressed values. The nature of these experimental data distributions dictates that the mean and median of the data can’t be equal. Indeed, as shown on Figure 1, the Mean/Median ratio for the majority of the datasets differed from 1. This parameter is a quick and simple estimate of microarray data quality, and should be included in data quality control.

Data from different technological platforms have different levels of noise and probe intensity and direct comparisons could be misleading without first correcting for these issues. Quantile normalization [29] is a means of normalizing data distributions to an averaged distribution across all datasets. As expected (Figure 4), an average distribution of all datasets, showed pronounced peak of low-expressed genes, which can be fitted with normal distribution. Although quantile normalization changed expression values slightly to make them fit to an average distribution, such changes are gradual. If a gene is co-expressed with another gene, a slight change in expression level even of both genes will not change the fact of their co-expression. Quantile normalization may slightly influence calculation of Pearson product-moment correlation coefficient, a metric for estimating correlation between two vectors, although we do not anticipate it to be a major problem.

Each experimental platform may suffer from erroneous or artefactual expression measurements, such as negative values, or extremely high expression. Since gene expression can’t be negative, such values, if present, should be flagged. In the present study datasets that passed quality control did not have negative values, which does not guarantee their absence in other datasets that could be added to the total pool in the future. We observed extremely high expression measurements across several datasets (e.g., where one gene is several orders of magnitude higher than any other). Such outliers may negatively affect performance of quantile normalization by skewing the average distribution in an unpredictable manner. Therefore, we “floored” top 0.1% of the data to decrease outliers’ effect. Although this “flooring” did not noticeably affect co-expression analysis (data not shown) we feel this negligible data treatment is an important precaution against possible technology-related errors.

To our surprise, even raw data can be used for predicting gene ontology categories using a global meta-analysis of all available data [8], although precision and recall are slightly less than processed data. Closer examination of the data revealed that even in raw data, gene-gene co-expression patterns are nonetheless discernable. This is similar to considering gene ranks [36] which are also retained in the raw data. Re-scaling the data, however, allowed more precise predictions to be made, since common thresholds could be applied. Quantile normalization further increased recall/precision of the predictions, because more exact thresholds can be defined. This step is imperative for comparative meta-analysis of data subsets, where absolute level of expression among different conditions and platforms should be compared.

## Conclusions

In this work we addressed potential pitfalls and problems associated with microarray meta-analysis of large number of disparate microarray experiments [22] and present specific steps and precautions. As such, the current paper aims at providing a means for creating a global, unified and directly comparable matrix of expression values associated with individual microarray experiments. We provide a framework for researchers to use pre-processed datasets of interest for their own research.

## Competing interests

The authors declare that they have no competing interests.

## Authors' contributions

JDW conceived of the project. MGD designed, implemented and tested the approach to normalization. Both authors wrote the manuscript. All authors read and approved the final manuscript.
